# Visual discrimination of polymorphic nestlings in a cuckoo-host system

**DOI:** 10.1038/s41598-018-28710-5

**Published:** 2018-07-09

**Authors:** Alfredo Attisano, Nozomu J. Sato, Keita D. Tanaka, Yuji Okahisa, Ralph Kuehn, Roman Gula, Keisuke Ueda, Jörn Theuerkauf

**Affiliations:** 1 Museum and Institute of Zoology Polish Academy of Sciences, Wilcza 64, 00-697 Warsaw, Poland; 2 Japan Bird Research Association, 1-29-9, Sumiyoshi-cho, Fuchu, Tokyo, 183-0034 Japan; 3 Department of Psychology, Keio University, Room 111, East Annex, 2–15–45, Mita, Minato, Tokyo, 108–0073 Japan; 4 Department of Life Sciences, Rikkyo University, 3-34-1 Nishi-Ikebukuro, Toshima, Tokyo, 171–8501 Japan; 5 Unit of Molecular Zoology, Chair of Zoology, Department of Animal Science, Technische Universität München, Hans-Carl-von-Carlowitz-Platz 2, 85354 Freising, Germany; 6 Department of Fish, Wildlife & Conservation Ecology and Molecular Biology Program, New Mexico State University, Box 30003, MSC 4901, Las Cruces, NM 88003-8003 USA

## Abstract

Mimicry by avian brood parasites favours uniformity over variation within a breeding attempt as host defence against parasitism. In a cuckoo-host system from New Caledonia, the arms race resulted in both host (*Gerygone flavolateralis*) and parasite (*Chalcites lucidus*) having nestlings of two discrete skin colour phenotypes, bright and dark. In our study sites, host nestlings occurred in monomorphic and polymorphic broods, whereas cuckoo nestlings only occurred in the bright morph. Irrespective of their brood colour, host parents recognised and ejected parasite nestlings but never ejected their own. We investigated whether host parents visually recognised their own nestlings by using colour, luminance and pattern of multiple body regions. We found that the parasite mimicked multiple visual features of both host morphs and that the visual difference between host morphs was larger than the difference between the parasite and the mimicked host morph. Visual discrimination alone may result in higher chances of recognition errors in polymorphic than in monomorphic host broods. Host parents may rely on additional sensorial cues, not only visual, to assess nestling identity. Nestling polymorphism may be a trace of evolutionary past and may only have a marginal role in true-recognition of nestlings in the arms race in New Caledonia.

## Introduction

The interactions between brood parasites and their hosts are a classic example of a co-evolutionary process in which adaptation on one side leads to counter-adaptation on the other and so on^1^. Phenotypic polymorphism is an example of such an adaption with an important role in the host-parasite co-evolutionary arms race^2^. For example, European Cuckoo *Cuculus canorus* females have two alternative plumage phenotypes that can help evading detection by the host^3–5^, while polymorphism in egg appearance may allow hosts to escape brood parasitism^6,7^. The evolution of successful host defences against brood parasitism, which involve the ability to detect and discriminate the brood parasite, is the fundamental driver in the arms race^1^. Thus, cognitive and perceptual abilities of the host are essential in the evolution and maintenance of phenotypic variation in both the host and parasite^2^. Variation in egg colour and pattern is an example of such defence strategy. In response to egg discrimination by hosts, brood parasites evolve egg mimicry^8–12^, which in turn favours the evolution of distinct egg signatures^13–15^ or distinct polymorphism in host clutches^6,7,16,17^. However, in a given host population, the success of such a defence strategy depends on phenotypic variation among clutches, which makes it difficult for the parasite to target a particular egg type, and on egg uniformity within clutches, which allows host parents to better discriminate a parasite against their own egg template^18–23^.

In some host-cuckoo systems the arms race has reached the nestling stage, and host parents discriminate parasite nestlings from their own^24–33^, which led to improved mimicry by cuckoo nestlings^34,35^ (but see^36^). In theory, the same mechanisms that determine egg polymorphism should also apply to nestling polymorphism^2^ and a recent study showed that polymorphism does indeed occur at the nestling stage in a cuckoo-host system in New Caledonia^37^. In this Pacific island, the local subspecies of the Fan-tailed Gerygone, *Gerygone flavolateralis flavolateralis*, is the exclusive host of the local subspecies of the Shining Bronze-cuckoo, *Chalcites lucidus layardi*. Mimicry by bronze-cuckoo nestlings is common in *Chalcites–Gerygone* systems^34,38^ and it also occurs in New Caledonia^37^. Despite the mimicry, two Australian *Gerygone* species^25,26^ and the Fan-tailed Gerygone from New Caledonia^37^ are able to recognise and eject the cuckoo nestling from their nest before it can evict any host egg or nestling.

In contrast to Australian *Gerygone* species, Fan-tailed Gerygone have two distinct nestling morphs, with a pinkish-grey (bright) or a darkish-brown (dark) skin, which can occur in monomorphic and polymorphic broods^37,39^. Brood colouration can vary within broods of the same parents, however it is most often constant for the same pair across multiple breeding attempts and extra-pair copulations do not influence the proportions of bright, dark or polymorphic broods^39^. The Shining Bronze-cuckoo from New Caledonia also has two nestling morphs, dark and bright^37^. Two nestling morphs, in this case however yellow and black, are also known to occur in the Australian Shining Bronze-cuckoo subspecies *C. l. plagosus*^34^. In general, nestling polymorphism is rare in birds^40^ and its occurrence within a brood parasite-host arms race is likely the result of strong selective pressure. Polymorphism in Shining Bronze-cuckoo nestlings may be an adaptation to mimic different hosts^34^, whereas the two host nestling morphs may have originated as a host defence strategy caused by the strong selection imposed by parasitism.

Polymorphic broods in a cuckoo host increase phenotypic variation and contradicts the principle of intra-nest uniformity^23^. This in theory should increase the likelihood of recognition errors by host parents. Within monomorphic broods, a cuckoo nestling might be accepted only if it matches the host nestling appearance and ejected if it does not, whereas in polymorphic broods a cuckoo nestling might always be accepted, because it matches one or the other host nestling morph. Moreover, in non-parasitised polymorphic broods, both host nestling morphs might sometimes be mistaken for a parasite and ejected from the nest. If nestling appearance is a cue for the recognition of the parasite, then polymorphic broods should decrease host fitness due to high chances of recognition errors. In our study sites, we only encountered the bright cuckoo morph and Fan-tailed Gerygone parents recognised and ejected the cuckoo nestling in both bright and dark monomorphic broods^37^. However, in our former research^37^, we were not able to confirm ejection of the parasite in polymorphic broods because none of the polymorphic broods in the previous study was parasitised.

It is also unclear how Fan-tailed Gerygone parents recognise the parasite nestlings. Some hosts of bronze-cuckoos discriminate the begging calls of the parasite from their own nestlings^24,29^. Although vocal learning can start in the pre-hatching period^29^, host parents can only discriminate nestlings once they emit begging calls with a definite structure, which usually forms around the age of 3–4 days^24,29,38,41^. Fan-tailed Gerygone host parents usually eject the parasite within 24 hours from hatching^37^ thus a similar auditory recognition based on properly structured nestling begging calls seems unlikely. Visual cues may be the main source of information for recognition. Fan-tailed Gerygone nestlings are covered by a dense layer of white multi-barbed down distributed over the head, back, rump, thighs and side areas, whereas Shining Bronze-cuckoo nestlings from New Caledonia have a sparser bristle-like down over the head, rump, thighs and side areas plus two dense layers of short white multi-barbed down around the orbital area^37^. The bristle-like down is unusual for parasite cuckoo nestlings^42^ and absent in the Australian Shining Bronze-cuckoo subspecies^34^, but bronze-cuckoos species that parasitise *Gerygone* hosts may present sparse down over some body areas^26,34,43^.

In this study, we investigated if visual cues are sufficient for host parents to obtain reliable information regarding nestling identity. Visual recognition of nestlings in the New Caledonian cuckoo–host system is a challenging cognitive task because host parents must recognise a parasite in either a monomorphic or a polymorphic brood and recognise the two host nestling morphs in any brood as their own offspring. Thus, visual cues alone may allow nestling recognition in all type of broods if, based on one or multiple visual features, the two host nestling morphs appear more similar to each other than to a parasite nestling. On the other hand, visual cues alone may not be sufficient to avoid recognition errors if, based on one or multiple visual features, the two host morphs appear more different to each other than to a parasite nestling. In this case, the recognition of the parasite may involve other sensorial cues, for example auditory or olfactory, or a combination of multiple sensorial cues.

We analysed the visual information available to Fan-tailed Gerygone parents from newly hatched parasite and host nestlings. This represents the time when the ejection occurs under natural conditions (usually within 24 hours from hatching). We quantified skin colour and luminance, nestling dorsal region colour and luminance (with skin and down combined as a unique visual stimulus), colour and luminance of rictal flanges and the influence of down on nestling visual pattern and contrast. We then compared the measured values between host morphs and cuckoo nestlings and between bright and dark host morphs. Finally, we quantified age-related changes in skin luminance of the two host nestling morphs by measuring skin luminance from hatching until the appearance of juvenile plumage. The main objectives of our study were to (1) verify that ejection of the parasite occurs in any type of gerygone brood, (2) assess the visual mimicry of the host morph by cuckoo nestlings, (3) quantify the effect of down on the overall colour, luminance, pattern and contrast compared to bare skin areas, (4) identify which visual cues may convey “host” vs. “non-host” information and (5) measure changes in skin colouration of host nestlings over time.

## Methods

### Study area and field methodology

We conducted field work at four sites on the main island (Grande Terre) of New Caledonia during six breeding seasons (September-January) in 2011/2012-2015/16 and 2017/2018: Parc provincial des Grandes Fougères (main study site) and near surroundings (21°37′39.44′′ S, 165°45′41.75′′ E), approx. 40 km west (21°35′58.89′′ S, 165°23′55.61′′ E) of the main study site, approx. 90 km southeast (22°9′36.96′′S, 166°25′24.11′′ E) of the main study site and approx. 130 km northwest (20°41′45.55′′ S, 164°59′′38.41′′ E) of the main study site. Field sites included areas of tropical rainforest (main study site) and savannah composed of grasslands and small patches of secondary forest (other sites). We located Fan-tailed Gerygone nests by following host parents returning to their nests. We found 190 active (containing at least one egg) nests of Fan-tailed Gerygones of which 30 contained cuckoo eggs, resulting in an overall parasitism rate of 16%. Is unlikely that the parasitism rate was influenced by early ejection of the cuckoo egg as the Fan-tailed Gerygone always accepted cuckoo eggs during our study, similar to other *Gerygone* hosts^44,45^. Upon finding an active nest, we monitored egg development by candling until the day of hatching. Due to high predation rates, we enclosed nests with eggs older than 7 days in chicken-wire fences, which allowed host parents to pass through, but kept the nest out of reach of the main avian predators. We took photos of nestlings on the day of hatching (defined as day 0). Fan-tailed Gerygone parents rarely abandon their own nestlings but may abandon an empty nest. Therefore, we always removed one nestling at a time from the nest. In case of a single nestling, one person stayed at the nest to prevent parents returning to the empty nest while another took the photos. No pair abandoned an active nest at the nestling stage due to this manipulation. We limited handling of nestlings to 3 minutes and returned gerygone nestlings to their nest immediately after taking the photos.

Because host parents usually ejected cuckoo nestlings soon after hatching^37^, we replaced the cuckoo eggs of 8 nests with model eggs resembling size and colour of the real cuckoo eggs. We then artificially incubated the cuckoo eggs and took photos of the hatchlings. We returned the artificially incubated cuckoo hatchlings into their nests of origin and video recorded the behaviour of host parents. If the original nest was predated, we introduced the cuckoo hatchling into a suitable active non-parasitised Fan-tailed Gerygone nest and recorded the behaviour of host parents. In these cases, we introduced an artificial cuckoo egg in the non-parasitised nest a few days before and successively exchanged it with the cuckoo hatchling. Artificially incubated cuckoo hatchlings were always returned to or introduced in the host nests on the day of hatching.

### Nestling photos

During four field seasons (2013/14, 2014/15, 2015/16, 2017/18), we took photos of gerygone and cuckoo nestlings on the day of hatching. The two Fan-tailed Gerygone nestling morphs are easily distinguishable at hatching, thus we categorised them as bright or dark by visual inspection, whereas we only found cuckoo nestlings of the bright morph in our field sites. Nests of Fan-tailed Gerygone are dome shaped and with a narrow entrance, which restricted the possibility of taking standardised photos of the nestlings inside the nest. We therefore placed the nestlings in a shaded area outside the nest to avoid heat shock by direct sunlight exposure and took the photos using natural illumination. Each nestling was placed in a standardised natural resting position into a rectangular open box (50 × 20 × 10 mm) padded with cotton and lined with a grey photographic mat background (ca. 18% reflectance). We placed the camera approximately 40 cm vertically above the nestling to include head, nape, back and rump areas. Each photo included a 98% optical PTFE white reflectance standard (Ocean optics WS-1). During the 2013/14 and 2014/15 field seasons we used an Olympus E-5 digital camera equipped with a Zuiko 35 mm macro lens and took photos using standardised settings (shutter priority mode, ISO 200, 1/60). We used these photos for visual analysis of nestlings in the human visible (VIS) light spectrum (400–700 nm). During the 2015/16 and 2017/18 field seasons, we took photos with a Fuji IS-PRO ultraviolet (UV) sensitive digital camera equipped with a Coastal Optic 60 mm UV macro lens (Coastal Optic Systems). We took ultraviolet photos using a UV pass filter (Baader UV filter, transmitting between 320 and 380 nm), and VIS photos using a UV and infrared (IR) cut filter (Hoya UV & IR cut filter, transmitting between 390 and 700 nm). A custom-built filter holder allowed quickly swapping UV and VIS filters on the camera lens between shots of each nestling. We used a Nikon SB-80DX flashlight unit with the UV filter removed when taking UV photos. This provided additional ultraviolet light, decreased the exposure time for the UV photos, avoided blurred images and decreased manipulation time of nestlings. We used standardised camera settings for the Fuji IS-Pro in all photos (VIS: ISO 100, f8, variable shutter speed in aperture priority mode; UV: ISO 100, f8, 1/125).

### Colour, luminance and pattern analyses

We linearised the red, green and blue camera sensor responses, standardised the digital photos in respect to light intensity using the pixel values of the 98% white standard and transformed pixel values to reflectance values^46^. Over-exposed or not correctly linearised photos were excluded from the analysis. We aligned, scaled and combined images from 2015/16 and 2017/18 to multispectral images including the UV and VIS visual information. Based on the spectral sensitivities of the Fuji IS-Pro UV camera, we converted the multispectral images from camera colour space to relative photon catches of avian shortwave (SW), mediumwave (MW), longwave (LW) and UV sensitive cone photoreceptors. Both the Shining Bronze-cuckoo and the Grey Warbler *Gerygone igata* from New Zealand, a close relative of the Fan-tailed Gerygone from New Caledonia, have a violet sensitive (VS) visual system^47^. We therefore used the spectral sensitivity of the most commonly used violet sensitive bird model, the peafowl *Pavo cristatus*^48^.

Following the methodology described by Troscianko & Stevens^49^, we generated polynomial models that translated the camera sensor response into peafowl cone-catch quanta by comparing the predicted camera and peafowl responses to a list of thousands of natural reflectance spectra. From calibrated multispectral images, we selected four regions per each focal nestling using the polygon selection tool in ImageJ^50^. We selected each region to include the most evident visual features of the nestling that host parents might use to recognise nestlings. For each region, we generated an average photon catch value (amount of photons that reach the retina) according to the cone response of the peafowl visual system. This allowed us to quantify how Fan-tailed Gerygone host parents perceive the visual features of nestlings.

We selected two regions of bare skin from head and rump of the focal nestling and averaged the values to a single ***skin*** value. The third region, ***dorsum***, included the entire dorsal region of the nestling in a natural resting position and thus combined bare skin and down. The fourth region, ***flanges***, included the lateral rictal flanges of the focal nestling. We measured colour and luminance of all selected regions, but measured pattern differences using only the ***skin*** and ***dorsum*** regions. We then compared these values between pairs of nestling types (bright host, dark host, parasite). We performed the skin colour and luminance analysis on multispectral images of 22 nestlings (15 bright host, 4 dark host, 3 parasite). Colour differences were calculated using the log version of the Vorobyev – Osorio receptor noise model^51^, which generated “just noticeable differences” (JND) values. This predicts whether two colours are discriminable in sufficiently good light conditions based on the signal-to-noise ratio of the focal visual system. A JND value below 1 means two colours are not discriminable, values between 1 and 3 mean two colours are difficult to discriminate except under optimal viewing conditions, values above 3 mean colours are increasingly easier to discriminate.

We measured achromatic (luminance) differences as described in Siddiqi *et al*.^52^. We calculated Weber fractions from the single cone ratios of the peafowl visual system (1: 1.9: 2.2: 2.1)^48^ and used a noise-to-signal ratio of 0.05 for the most abundant cone type. We compared each individual nestling with all nestlings of a different type to obtain three groups of JND values: Cuckoo-Bright (cuckoo nestlings compared to the bright gerygone morph), Cuckoo-Dark (cuckoo nestlings compared to the dark gerygone morph) and Bright-Dark (bright compared to dark gerygone morph). Thus, our analysis included absolute comparisons of nestling pairs (JND values for each group) and relative differences between groups (JND comparisons between groups) in monomorphic and polymorphic host broods.

To measure the effect of down on the visual appearance of nestlings compared to bare skin areas, we used pattern difference analysis. We used VIS photos of 78 nestlings: 8 cuckoo, 59 bright and 11 dark host. We linearised images and converted them to reflectance values (RGB-equalised). However, we did not convert them into the predicted response of the peafowl visual system because we did not know the sensor spectral sensitivity of the Olympus camera. We thus obtained measurements as camera response objective values in the VIS spectrum (400–700 nm). We followed the Fourier analysis and bandpass filtering approach used in previous studies of animal markings and patterns^53,54^. We generated pattern differences using a Fast Fourier Transform bandpass filter at 17 levels (from 2 pixels, increasing exponentially with √2 up to 350 pixels) and calculated spatial frequency differences between the two focal nestlings at each spatial scale by summing the absolute difference in energy. Such pattern difference describes the degree to which the nestling patterns match each other in terms of size, spacing and contrast. We used the same three groups as in the colour and luminance analysis to obtain the absolute comparison between pairs of nestlings (pattern difference value for each group) and relative differences between groups (pattern difference comparisons between groups).

In 2014/15 and 2015/16, we took photos of the ventral side of the focal nestling each day from day 0 (hatching day) until day 11. We chose the ventral side of the focal nestlings because it was featherless until the age of 13–14 days and thus allowed us to isolate skin areas to follow changes in skin luminance. We collected photos from 73 bright and 17 dark host nestlings. As we took photos with two different cameras, we only used objective reflectance measurements in camera colour space and linearised and standardised the photos relative to the 98% white standard. Markings and patterns are mainly encoded by achromatic information, thus we used the green camera sensor as this more closely corresponds to the avian luminance channel^55^. We then converted measured reflectance values into proportions relative to the 98% white standard.

### Statistical analyses

We ran mixed models to analyse the difference in colour JND, luminance JNDs and pattern difference within each region (***skin, flanges, dorsum***). We used GLMMs with a log link function to analyse colour and luminance JNDs and a LMM to analyse the pattern difference. We applied log transformation of the pattern difference values to achieve normally distributed residuals. The models included comparison (Bright-Dark, Cuckoo-Dark, Cuckoo-Bright) as main effect and site and pair ID as crossed random effects. We did not have cases of multiple breeding by the same pair (in the same or different years) in the used data set. Upon finding a significant effect, we ran a post-hoc pairwise comparisons between pairs of groups within each region. We quantified the rate of change in luminance during development of the two host morphs with an LMM for repeated measures that included site, nestling age, skin colour and the interaction age*skin as fixed effects and nestling ID (nested within age) as random effect. We used the Image Analysis Toolbox in ImageJ^49^ to prepare the multispectral images, convert them into cone catches and obtain colour and luminance JNDs and pattern difference values. All statistical analyses were run in R 3.2.1^56^.

### Data availability

All data generated or analysed during this study are included as Supplementary Information files.

### Ethical statement

The Province Sud of New Caledonia issued all permits (3045-2011, 2437-2012, 2532-2013, 2801-2014, 2476-2015, 2372-2017). Handling and collection of the nestlings was performed in accordance with the guidelines and regulations outlined in the permits. The 1^st^ Warsaw Local Ethics Committee for Animal Experimentation approved the experimental procedures.

## Results

### Ejection of the parasite

In 30 parasitised breeding attempts, 15 cuckoo nestlings hatched, whereas in the other 15 cases the cuckoo did not hatch (3 cases), predators destroyed the nest (8) or we could not follow the nest until hatching (4). In all 15 cases of cuckoo hatching, the host parents ejected the parasite within 24 hours from hatching in all brood types (Table 1). We never observed Fan-tailed Gerygone host parents ejecting a Shining Bronze-cuckoo egg or one of their own nestlings in any brood type. We observed 9 cases of nest desertion at the egg stage (5% of 190 active nests) in 8 unparasitised nests and in 1 parasitised nest. The unparasitised nests were deserted in 7 cases due to unintended disturbance caused by the nest checks or placing of the video cameras and once after disturbance by predators. The parasitised nest was deserted 1 day after host parents ejected the cuckoo nestling, most likely as a consequence of human disturbance (placement of the protective fence) rather than parasitism. Thus, none of the nest desertion we observed seemed to be a direct consequence of brood parasitism, but rather an involuntary consequence of our research activities.

**Table 1:** Total number of Fan-tailed Gerygone broods, nests parasitised by Shining Bronze-cuckoo, number of cuckoo hatchlings and number of parasite nestling ejection events observed during the period 2011/12-2015/16 and 2017/18.

Brood type	Number of broods	Parasitised nests	Cuckoo nestlings	Ejection events**
Bright	66	7	7	7
Dark	13	3	3	3
Polymorphic	8	1	1	1
unknown brood type*	104	19	4	4
	191	30	15	15

*We could not determine the brood phenotype due to predation, hatching failure or accidental damage of the eggs. **In 11 naturally parasitised nests (5 bright monomorphic broods, 2 dark monomorphic, 1 polymorphic and 3 broods of unknown nestling composition) and in 4 nests (2 bright, 1 dark and 1 unknown) to which we introduced artificially incubated cuckoo nestlings.

Successful parasitism was too rare to be detected with only 30 parasitised and monitored breeding attempts, which means that in our sites the parasite success should be lower than 3% (i.e. 1/30). We however observed four cases of successful parasitism at other sites: one observation of a cuckoo nestling (about 11 days old) and three observations of a cuckoo fledgling fed by both Fan-tailed Gerygone host parents. In none of these four cases, we could determine the original host brood phenotype or if the cuckoo nestling had evicted host eggs or nestlings. Cuckoos hatched earlier than host eggs but gerygone parents did not always eject the cuckoo nestling before their own offspring hatched. Thus, Shining Bronze-cuckoo nestlings coexisted with Fan-tailed Gerygone nestlings in 6 broods (4 bright, 2 dark and 1 polymorphic) before being ejected by host parents.

### Colour and luminance differences

**Figure 1:**
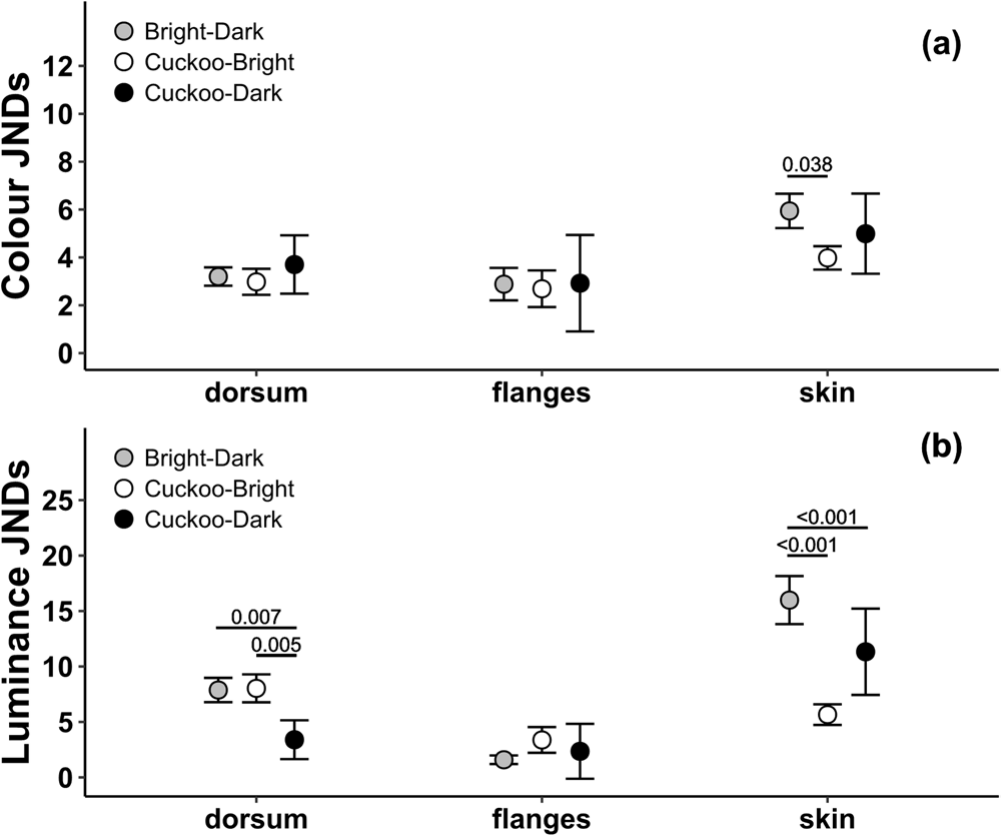
Colour (**a**) and luminance (**b**) just noticeable differences (JND, mean ± 95% CI). Horizontal bars with P values indicate pairwise comparisons between groups or regions. Higher JND values indicates higher colour differences in the eyes of host parents between nestling types in each group. Sample size (number of comparisons) for ***dorsum*** and ***skin*** regions are Bright-Dark = 60, Cuckoo-Bright = 45, Cuckoo-Dark = 12 and for the ***flanges*** region are Bright-Dark = 27, Cuckoo-Bright = 18, Cuckoo-Dark = 6.

Cuckoo nestlings mimicked the colour of both host morphs (Fig. 1a). The bright and dark host morphs had a relatively larger colour difference in the ***skin*** region than the cuckoo and both host morphs (Fig. 1a). All nestling groups had similar colour differences in the ***dorsum*** and ***flanges*** regions (Fig. 1a). The colour of the ***skin*** region was generally discriminable among nestlings in good light conditions whereas mean colour JND values were around the discrimination threshold of 3 JNDs for all nestling groups in the ***dorsum*** and ***flanges*** regions (Fig. 1a). The presence of down decreased the colour differences compared to bare skin areas (***dorsum*** vs. ***skin***, mean JNDs ± 95% CI: 3.16 ± 0.30 vs. 5.09 ± 0.46).

Cuckoo nestlings mimicked skin luminance of both host morphs, in particular the bright morph, whereas the bright and dark host morphs had a relatively larger skin luminance difference than the cuckoo compared to both host morphs (Fig. 1b). Cuckoo nestlings were also more similar to dark host morphs in the ***dorsum*** region (Fig. 1b). Down of nestlings decreased achromatic differences compared to bare skin areas (***dorsum*** vs. ***skin***, mean JNDs ± 95% CI, 7.48 ± 0.79 vs. 11.54 ± 1.49). There was no difference between nestling groups in the ***flanges*** region (Fig. 1b). With the exception of the ***flanges*** region, all mean luminance JND values were above the discrimination threshold of 3 JNDs for all groups within each region.

**Figure 2:**
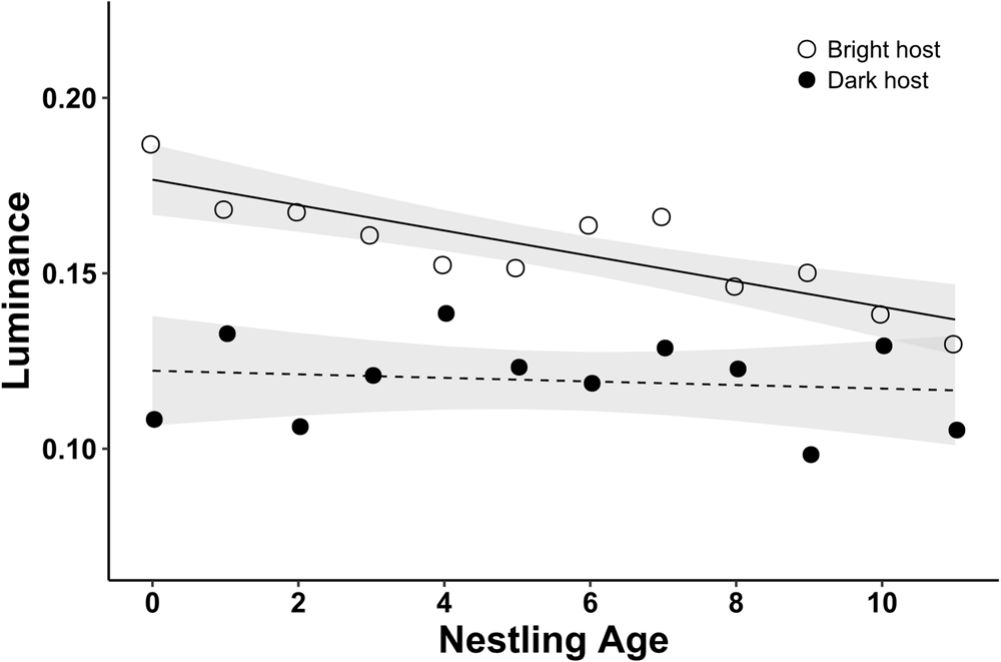
Regressions lines (with 95% CI as shaded area) of skin luminance in relation to age for the bright (solid line) and dark (dashed line) morphs of Fan-tailed Gerygone nestlings.

Skin of bright Fan-tailed Gerygones darkened as the nestlings grew older, whereas the dark nestlings kept their luminance (Linear mixed model, age*skin, n = 90, Wald χ^2^ = 5.32, d.f. = 1, *P* = 0.021; Fig. 2).

### Pattern differences

**Figure 3:**
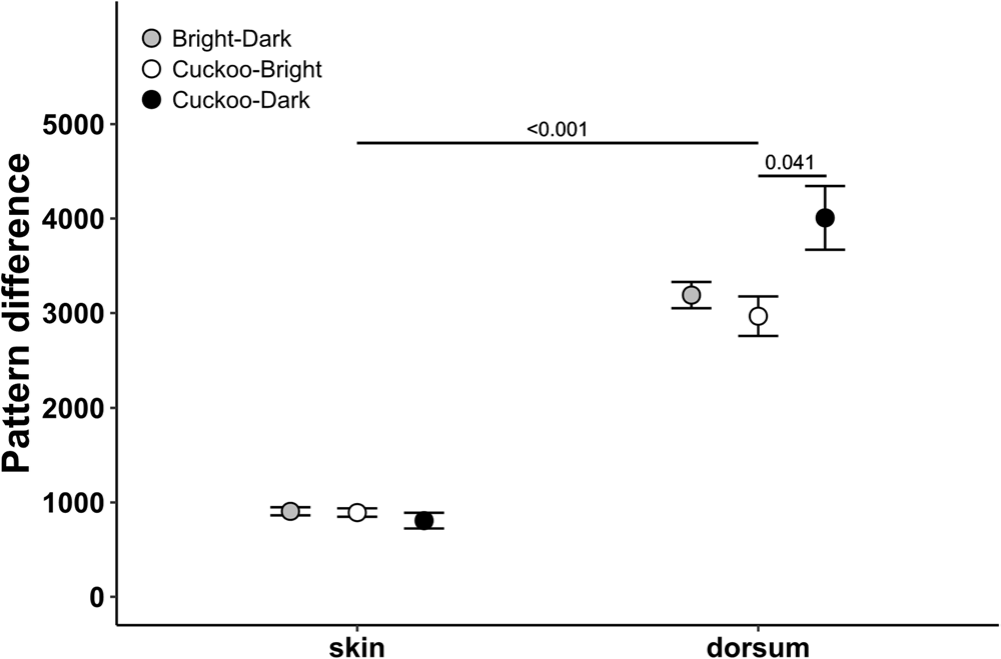
Mean pattern differences (pattern size, spacing and contrast) with 95% CI between nestling types in each group for each region of interest. Horizontal bars with P values indicate pairwise comparisons between groups or regions.

The presence of down increased the pattern difference between nestlings in the ***dorsum*** region compared to the bare areas of the ***skin*** region (Fig. 3). Within the ***dorsum*** region, cuckoo chicks had lower pattern differences with bright host morphs than with dark host morphs (Fig. 3). Moreover, bright host morphs had a similar pattern difference with cuckoo chicks and dark host morphs (Fig. 3). There was no pattern difference between groups in the ***skin*** region (all pairwise comparisons *P* > 0.1; Fig. 3).

## Discussion

We found that visual mimicry by Shining Bronze-cuckoo nestlings from New Caledonia was based on the mimicking of multiple visual features of the host, similar to bronze-cuckoos from Australia^34,42^. We expected an improved mimicry of the respective similar host morph, i.e. bright cuckoo nestlings would better mimic bright host nestlings and dark cuckoo nestlings would better mimic dark host nestlings. However, we found only a bright cuckoo morph with features that improve mimicry with both host morphs rather than expressly mimicking only the bright host morph. The most conspicuous of these features is the presence of the white down. Host parents may use these features to visually recognise their nestlings, but cuckoo nestlings might also evade detection by the host because they are more similar to either host morphs when down and skin are combined as a single visual stimulus. More specifically, the down makes bright cuckoo nestlings more similar to both host morphs in terms of colour and more similar to dark host morphs in terms of luminance. The down also creates a high visual contrast with bare skin areas that increases the cuckoo mimicry with the bright host morph, whereas the dark morph has a higher contrast difference with both the cuckoo and the bright host morph. Similar to other brood parasites^57,58^, cuckoo nestlings mimic the colour and luminance of the rictal flanges of host nestlings, as these are almost completely indistinguishable among all nestlings.

Although the Shining Bronze-cuckoo from New Caledonia parasitises a single host, it seems to have a mimicry strategy comparable with a generalist brood parasite. Its bright nestlings have features that closely resemble the preferred host, in this case the more frequent bright morph, but also general features that allow exploiting a “secondary host”, in this case the dark morph of the same host species. Generalist brood parasites exploiting different hosts are known to employ “average appearance” strategies. For example, genetic races (gentes) of the Common Cuckoo mimic eggs of their favourite hosts but also have “average” egg appearance that can facilitate the use of secondary hosts^53^. Similarly, the generalist Horsfield’s Bronze-cuckoo *Chalcites basalis* lays eggs that are intermediate in appearance with the eggs of its various hosts^59^. Nestlings of the Horsfield’s Bronze-cuckoo also have a skin colouration that facilitates mimicry of their various host nestlings^34^. The Shining Bronze-cuckoo subspecies from Australia, *C. l. plagosus*, has two nestling morphs that may depend on the presence of gentes specialised on particular hosts^34,43^. In New Caledonia, the occurrence of possible gentes of the Shining Bronze-cuckoo subspecies *C. l. layardi* with bright or dark nestlings might depend on local frequencies of the host phenotypes. The relatively high frequency of bright host broods in our study sites may thus have led to low frequencies, or even disappearance, of the dark cuckoo morph which would explain why we never found a dark cuckoo morph during our study. In this case, there would only be selection acting on an average appearance of the bright cuckoo morph rather than on the frequencies of two contrasting cuckoo morphs.

The two host nestling phenotypes differ from each other in skin colour and luminance. In most cases, the dark host morph was as different from the cuckoo nestling as it was from the bright host morph. Luminance of the rictal flanges appeared to be the only feature with low visual difference between the two host morphs thus offering the only visual signals that could potentially allow distinguishing host from parasite. However, the use of the rictal flanges as a discriminatory cue may prove unreliable as the comparisons of colour and luminance between host and parasite nestlings showed low or no differences, meaning that cuckoo nestlings may exploit this visual signal to their advantage. Despite the striking visual difference between the two host morphs, we confirmed that ejection of the parasite occurred in the two monomorphic and in the polymorphic broods, extending previous observations on the ejection behaviour in Fan-tailed Gerygone^37^. A single host-specific visual signal is unlikely to be a reliable discriminator between “host” and “non-host” as none of the visual signals taken singly may offer certainty of reliable recognition. Rather, host parents must combine multiple visual features of the nestlings to achieve discrimination of the parasite. Nevertheless, the recognition of the parasite by host parents is easier in monomorphic broods. In this case, cuckoo nestlings should be accepted 1) if all visual features are perfectly matched or 2) if host parents have low discrimination ability for one or multiple visual features. In polymorphic broods, the recognition process is far more complex because in parasitised broods, the dark host morph is the odd-looking one and thus it should more likely be recognised as “non-host”. Moreover, in non-parasitised polymorphic broods, the two host nestling morphs appear different from each other, thus one morph should theoretically be recognised as “non-host” and ejected. However, this did not happen during our research. This raises the question of how host parents identify both host morphs as their own offspring and do not eject one as “non-host” by mistake.

Several studies showed that egg ejector hosts are able to recognise the parasite by discordancy with the majority of the clutch. For example, host parents may compare size^60^ or colour and pattern^61–63^ of a foreign egg against the majority of the eggs in the clutch. Alternatively, host parents can recognise the parasite via true-recognition by comparing eggs to a learned template^64,65^. In some cases, the two strategies can be used in conjunction^66^. A recognition by discordancy seems unlikely in Fan-tailed Gerygone because of its low average clutch size of 2 eggs^37^ and because Shining Bronze-cuckoo females usually remove one of the host eggs before laying their own^67^. In an average parasitised Fan-tailed Gerygone brood, the ratio between host and parasite is therefore 1:1, which makes it impossible to compare one nestling against a majority. In addition, because the two host morphs greatly differ from each other, a recognition by discordance may lead to the ejection of one of the host nestlings in polymorphic broods despite the presence of the parasite. Our observations that ejection of the parasite can occur before hatching of host nestlings suggest that Fan-tailed Gerygone parents achieve true-recognition via a comparison with a cognitive template. The true-recognition of the parasite in Fan-tailed Gerygone is thus a more refined behaviour than other forms of advanced discrimination described in various cuckoo hosts^24,28,36^. True-recognition and ejection of a parasite mimicking host nestlings requires high cognitive abilities from host parents, which may explain why such host defence behaviour is rare^33^. It will take further research to find out whether the template is innate or learned in Fan-tailed Gerygone parents (see^24,41^).

Although visual cues may have an important role in the parasite recognition process, their reliability may be limited by low light conditions in the incubation chamber of dome-shaped nests compared to open cup nests^68^. Thus, the true-recognition by Fan-tailed Gerygone host parents might be based on a combination of multiple sensorial cues. Auditory cues may have an important role in the recognition of a foreign nestling. For example, Superb Fairy-wrens *Malurus cyaneus* can discriminate Horsfield’s Bronze-cuckoo nestlings by the different structure of their begging calls^24,41^. In addition, bronze-cuckoo nestlings mimic the begging calls of their main host^38,41^. However, nestlings may not produce proper begging calls until 3–4 days of age^38,41^, whereas Fan-tailed Gerygone host parents eject the parasite nestling within 24 hours from hatching. In addition, ejection can occur before hatching of the host nestlings thus host parents cannot always compare, or do not need to compare, begging calls of the parasite and host. Another possibility is that true-recognition involves olfactory cues. For example, European Magpies *Pica pica* can discriminate foreign eggs in their clutch via odour cues^69^ and experimental evidence seems to indicate that at least some birds may be capable of odour self-recognition^70^. Olfactory cues may be particularly useful in a dome-shaped nest where volatile compounds may concentrate in a restricted space. Low light conditions in the nest may thus favour the use of auditory and olfactory cues for discrimination when visual cues becomes unreliable.

Nestling polymorphism is a rare occurrence in birds^40^, thus the polymorphism of host and parasite in the New Caledonia system likely originated because of strong selective pressures imposed by the arms race. Bronze-cuckoo nestlings are known for their plasticity to mimic the preferred host^34^, similar to host-specific races of the Common Cuckoo mimicking eggs of different hosts in Europe and Asia^71^. On the other hand, host nestling polymorphism should rarely occur because it would increase the likelihood of committing recognition errors^23^. To our knowledge, only two cases of nestling polymorphism in hosts of brood parasites have been described and both involve a *Gerygone* host: the Fan-tailed Gerygone^37^ from New Caledonia and the Grey Gerygone^72^ from New Zealand. However, while the Fan-tailed Gerygone is a nestling ejector, the Grey Gerygone is neither an egg^44^ nor a nestling Briskie, pers. comm. ejector. Other nestling ejecting *Gerygone* species seem to have only monomorphic broods with either dark or bright nestlings^25,26^. In the Fan-tailed Gerygone, there are two possible scenarios to explain the colour of nestlings at hatching. In the first scenario, the dark morph is ancestral and a down-regulation mechanism of the melanocortin system suppresses the melanin synthesis with a consequent appearance of a bright morph at hatching. In the second scenario, the bright morph is ancestral and an up-regulation mechanism of the melanocortin system causes a dark morph to appear at hatching. A recent phylogenetic analysis^73^ shows that the Fan-tailed Gerygone is most closely related to *Gerygone* species with dark nestlings^26^, and in this study, we show that the bright host morph progressively darkens with age, whereas no change occurs in the dark morph. This suggests that the dark host morph is ancestral and that cuckoos probably mimicked it in the initial phases of the arms race. Mimicry by Shining Bronze-cuckoo nestlings then probably promoted polymorphism in the host with the appearance of a bright host morph that increased in frequency in the population under negative frequency selection. This in turn likely promoted polymorphism in cuckoo nestlings with the appearance of a bright cuckoo morph. Thus, host nestling polymorphism was probably favoured as host defence in the first stages of the arms race if the same evolutionary mechanisms as for egg polymorphism apply to nestling polymorphism^2^. However, egg phenotype solely depends on the mother^74^, whereas nestling phenotypes depend on the genetic background of both parents^75^. Thus, nestling phenotypes may change if parents exchange partners or incur in extra-pair copulations and this may strongly constrain the selective advantage of nestling polymorphism as a host defence. In the Fan-tailed Gerygone, extra-pair copulations had however no impact on the frequency of nestling or brood types^39^. Nevertheless, polymorphic broods may have further promoted the arms race, which involves generalist mimicry by the parasite and true-recognition based on multiple sensorial cues by the host.

## Electronic supplementary material

Dataset 1

Dataset 2

Dataset 3
